# Factors associated with digital tools use among primary healthcare professionals in Burkina Faso: a cross-sectional study of the minimal digital ecosystem

**DOI:** 10.1186/s12913-026-14331-6

**Published:** 2026-03-12

**Authors:** Relwendé Nacanabo, Joël Arthur Kiendrébéogo, Noëllie Konsebo, Issa Kaboré, Orokia Sory, David Zombré, Rémi Kaboré, Simon Tiendrebeogo, Charlemagne Tapsoba, T. Daliah Marie Nacoulma, Toudala Paré, Yamba Kafando

**Affiliations:** 1 Institut de Recherche en Sciences de la Santé (IRSS), Ouagadougou, Burkina Faso; 2 Recherche pour la santé et le développement (RESADE), Ouagadougou, Burkina Faso; 3 Université Joseph KI-ZERBO, Ouagadougou, Burkina Faso; 4 Institut de Santé Publique d’Épidémiologie et du Développement (ISPED), Bordeaux, France

**Keywords:** Digital health, Minimal digital ecosystem, Healthcare professionals, Tool use, Logistic regression, Health services research, Burkina Faso

## Abstract

**Background:**

The use of digital tools in healthcare remains limited in many countries, although their use by healthcare professionals is key to the effective implementation of digital health. In 2023, Burkina Faso introduced the Minimal Digital Ecosystem (MDE), a set of eight digital tools designed to support healthcare providers in clinical care, free-service management, pharmaceutical supply, and quality assessment. This study aims to identify factors associated with the use of MDE tools among healthcare professionals.

**Methods:**

We conducted a cross-sectional study in four districts of Burkina Faso (Ziniaré, Ténado, Manga, and Sapouy). Spatially representative health facilities were selected using Moran autocorrelation, and purposive sampling was applied to recruit health professionals. Data were analysed through chi-square tests and multivariate logistic regression.

**Results:**

Factors varied across tool categories. Use of service delivery tools (*REC-PCIME* and *REC-Maternité*) was significantly associated with secondary education (aOR = 3.47; 95% CI: 1.68–7.20). Perceived ease of use strongly influenced *E-SantéCom* adoption (aOR = 98.51; 95% CI: 1.77–5492.15). Men were more likely to use *NetSIGL 2.0* (aOR = 3.07; 95% CI: 1.76–5.36), while access to more than four tablets reduced its use (aOR = 0.56; 95% CI: 0.35–0.91). Geographic disparities were observed for *E-Qualité*, with almost no uptake in Manga (aOR = 0.01; 95% CI: 0.00–0.18). For *E-Flux financier*, secondary education was again a major predictor (aOR = 7.66; 95% CI: 1.45–40.38). Finally, use of *E-Gratuité* and individual electronic care record tool *(FIS)* was associated with male gender (aOR = 18.04; 95% CI: 5.14–63.30) and more than 10 years of professional experience (aOR = 12.05; 95% CI: 1.59–91.46).

**Conclusion:**

MDE tools use in Burkina Faso is shaped by both individual factors (gender, education, experience, and perceptions) and structural factors (equipment availability and geographic disparities). Targeted strategies are needed to strengthen digital health use and ensure equitable integration within primary healthcare.

**Supplementary Information:**

The online version contains supplementary material available at 10.1186/s12913-026-14331-6.

## Introduction

Digital health technologies have expanded rapidly in recent decades and are defined as the secure and cost-effective use of information and communication technologies to support health and related fields, including service delivery, health surveillance, patient education, and research [1]. A wide array of heterogeneous solutions now exists, differing in technological design, functionalities, and intended users such as patients, caregivers, or healthcare professionals [2]. Evidence shows that digital health can substantially improve the efficiency of healthcare systems by enhancing both service provision and system administration [3, 4]. For instance, electronic medical records can facilitate therapeutic planning and provide timely reminders for preventive and screening interventions [3]. More broadly, digital tools have been shown to significantly strengthen health system performance [5].

Despite these benefits, numerous challenges remain, especially in low-resource settings such as sub-Saharan Africa [5]. These include limited availability and maintenance of digital equipment, underdeveloped information technology infrastructure, and poor Internet accessibility [6]. Mobile phone penetration in sub-Saharan Africa was estimated at 44% in 2017, but mobile Internet access remained as low as 21% [7]. Furthermore, interoperability between digital systems is a recurrent obstacle, as many applications and databases operate in silos, hindering the integration of patient data and limiting providers’ ability to make informed decisions [8–10].

Although digital technologies hold the potential to transform health service delivery in resource-constrained contexts, their adoption and sustained use remain limited across many African countries [11–13]. Even with recent advances, utilisation rates are still low, and effective adoption by healthcare professionals is widely recognized as a key determinant for the successful implementation of digital health innovations [11, 14].

Burkina Faso has over a decade of experience with health digitalisation, driven by the National Health Information System (NHIS) strategic plan 2010–2020 and multiple initiatives supported by the Ministry of Health and non-governmental organisations [15]. Several digital solutions have been implemented at different levels of the health system. At the community level, the *E-SantéCom* application was introduced in 2016 to support community health workers in monitoring child care, training, and awareness campaigns [16]. In primary healthcare facilities, the electronic consultation register (*REC*) has been deployed since 2010, digitising the Integrated Management of Childhood Illness (*PCIME*) protocol and offering decision-support features, patient records, multimedia guidance, and e-learning modules [17, 18]. However, the REC has been hampered by technical constraints such as slow tablets, delayed data synchronisation in areas with poor connectivity, and limited resources for equipment renewal [19].

Beyond these technical hurdles, the national digital landscape has faced major structural and political obstacles. Over the last decade, political leadership was often undermined by a proliferation of vertical, donor-driven solutions that operated largely in silos. Parallel initiatives have produced a proliferation of digital platforms, including *Viamo*, *STElab*, *NetSIGL 2.0*, *E-Gratuité*, *E-Qualité*, and *E-Flux financier*. At hospital level, data management is highly fragmented, with multiple non-harmonised applications and limited interoperability [14]. In 2013, the Ministry of Health established the National Health Data Warehouse (ENDOS-BF) using DHIS2 to centralise data from different levels of the health system. Yet, despite its potential, interoperability challenges between ENDOS-BF and other applications persist, hindering the integration and use of health data [15]. This lack of coordination often fuelled by fragmented funding from diverse international partners prevented health authorities from having a unified and reliable view of system performance. By 2020, more than 110 distinct digital applications had been identified across the national health pyramid, many of which were either duplicates or no longer functional, reflecting a highly fragmented digital ecosystem [15].

In response to this fragmented landscape and the urgent need to rationalise limited resources, the Ministry launched the Minimal Digital Ecosystem (MDE) in 2023. Unlike previous vertical initiatives, the MDE represents a strategic national reform designed to integrate eight core applications, covering consultation, free healthcare management, pharmaceutical supply, financial flow management, and quality monitoring [20]. Focusing on the MDE is paramount because it constitutes the new digital backbone of primary healthcare. While this initiative aims to improve efficiency and service quality, evidence on the actual use of these tools by healthcare professionals remains scarce. Understanding the factors influencing its use is crucial, as the equitable and effective adoption of this integrated ecosystem by frontline workers is the primary driver for improving the overall efficiency and equity of Burkina Faso’s health system. The uneven utilisation of digital technologies raises important questions about the factors influencing their use. Existing studies highlight the influence of socio-demographic characteristics such as gender, age, education, and perceived usefulness [21–23]. However, few studies conducted in sub-Saharan Africa have quantitatively examined how individual, organizational, and contextual factors jointly influence the use of digital health tools at the primary care level [11, 14]. Drawing on established technological acceptance frameworks [21–23], we anticipated that a combination of individual, organizational, and contextual factors would influence the uptake of digital tools. These theoretical frameworks are complemented by empirical evidence from other developing countries, where education, gender, and perceived ease of use have been identified as key predictors of digital engagement [24–26]. Against this backdrop, this study seeks to identify factors associated with the use of MDE tools among healthcare professionals in four districts of Burkina Faso. Specifically, it aims to (i) profile users to understand variations in use, (ii) analyse associations between tool use, socio-demographic attributes, and health facility characteristics, and (iii) provide recommendations for improving the use of digital tools in primary healthcare.

In light of these elements, we made the following assumptions: (H1) higher education and longer professional experience would be associated with greater use of digital tools; (H2) male service providers would report greater use of digital tools than female service providers; and (H3) perceived ease of use, adequate training, and the level of deployment of digital tools would be positively associated with their use.

## Materials and methods

### Research framework

The intervention was carried out in four health districts of Burkina Faso: Ziniaré, Manga, Ténado, and Sapouy. Selection of these districts was guided by three criteria: (i) the level of security in relation to terrorism, (ii) geographical distance from the capital, Ouagadougou, and (iii) the urban or rural profile of the district. Ziniaré and Manga were chosen as districts comparable in population size and healthcare service utilisation. Ziniaré lies in close proximity to Ouagadougou, whereas Manga is located further south. Both districts presented relatively favourable conditions for the implementation of the Minimal Digital Ecosystem (MDE), including a higher and more stable availability of electricity and Internet connectivity across health facilities [27]. At the time of the study, Ziniaré and Manga had 81 and 50 public primary healthcare facilities, respectively [27]. By contrast, the districts of Ténado and Sapouy are characterised as rural or semi-urban settings with more limited access to energy and Internet services, thereby presenting less favourable conditions for digital health deployment [27]. Ténado counted 28 public healthcare facilities, while Sapouy had 34. For the purpose of statistical comparisons, Ziniaré and Manga were considered similar and analysed jointly, while Ténado and Sapouy were treated as comparable rural counterparts [27].

### Study design

This study employed a cross-sectional design and was conducted in four health districts of Burkina Faso (Ziniaré, Manga, Ténado, and Sapouy). This research is part of a broader longitudinal evaluation of the Minimal Digital Ecosystem (MDE), which includes five survey waves scheduled between 2023 and 2026; the present analysis specifically draws on the second wave conducted in June 2024. The MDE was initially launched in 2023 as a pilot phase in the districts of Ziniaré and Ténado. Subsequently, the intervention was expanded to Manga and Sapouy to align with evolving national digital health priorities. Notably, certain components, such as the *FIS* tool, were scaled up nationwide to support the government’s free healthcare policy. By the time of data collection in June 2024, the implementation of the eight MDE tools had reached a comparable and synchronised level across the four study districts, justifying the use of a cross-sectional approach to identify factors associated with their use. This design allowed for the assessment of statistical associations between the self-reported use of MDE tools and the sociodemographic characteristics of healthcare professionals, as well as organisational and infrastructural factors at the health facility level. 

### Description of the “minimal digital ecosystem” intervention

The MDE was introduced in Burkina Faso in 2023 as part of a national strategy to rationalise the fragmented digital health landscape, which had previously been characterised by more than one hundred disparate applications operating in silos [20]. The MDE consists of a suite of eight core digital solutions, implemented synergistically within healthcare structures ranging from community-based health workers to primary facilities (CSPS) and district-level referral facilities (CM and CMA). It is specifically focused on the primary healthcare level and does not extend to regional (CHR) or university (CHU) hospital centres. These applications include an electronic consultation register for childhood illness management (*REC-PCIME*) and a maternity register (*REC-Maternité*) to support maternal and child health services; financial management tools such as *E-Flux financier* and the Individual electronic care record (*FIS*); a pharmaceutical logistics platform (*NetSIGL 2.0*); a community health application (*E-SantéCom*) for referrals and health promotion; and quality assurance and resource-tracking systems (*E-Qualité* and *E-Gratuité*). Together with a central dashboard of indicators, these tools aim to enhance service delivery, improve the management of free healthcare policies, and strengthen accountability mechanisms. The strategic objective of the intervention is to increase the efficiency, quality, and equity of healthcare provision, thereby contributing to reductions in maternal and child mortality as well as in catastrophic household health expenditures. A more detailed description of these digital solutions has been documented elsewhere [20].

### Population studied

The study population consisted of a wide range of healthcare actors operating in different types of health facilities, exclusively health and social promotion centres (CSPSs) and medical centres (CMs). It included health facility managers, frontline care providers working within these facilities, and community-based health workers (CBHWs) from the villages covered by the selected CSPSs. Members of the management committees (*COGES*), in particular the chairpersons and treasurers, were also part of the study, along with administrative managers, cashiers, and the managers of depots for essential generic medicines (*DMEG*). Together, these groups represent the key stakeholders involved in both the delivery of primary healthcare services and the management of resources within the local health system.

### Sampling

The MDE intervention was designed to encompass all public primary healthcare facilities within a given district. Consequently, our study covered all public facilities providing PHC in the four selected districts. In consultation with the Ministry of Health, efficiency was identified as a key parameter for sampling because a core objective of the MDE is to rationalize fragmented digital systems and optimize health system performance. The cost of a normal vaginal delivery was retained as the critical indicator because it represents a standardised, high-volume service common to all CSPS, making it a reliable and sensitive proxy for facility-level operational efficiency and resource management. Using 2022 cost data, the estimated mean and standard deviation for a delivery were 4,600 FCFA (SD 900) in Ziniaré and 4,210 FCFA (SD 972) in Ténado. Notably, 73.6% of CSPSs in Ziniaré and 60.7% in Ténado exhibited mean delivery costs outside these expected ranges, which underscored the variability of service efficiency across facilities and guided the sampling frame. The minimum sample size of health facilities was determined using a formula based on the proportion of CSPS whose average cost of a normal vaginal delivery would fall outside predefined confidence intervals at the end of the intervention [27]. Parameters were set in consultation with the Ministry of Health, with a first-species error of 5%, a power of 80%, and a cluster effect of 2, as recommended.

The minimum size of CSPS required to demonstrate the difference ${p}_{2}-{p}_{1}$ is given by the following formula [28]:

$$n=f\cdot\frac{{\left({Z}_{\alpha}+{Z}_{\beta}\right)}^{2}\cdot\left[{p}_{1}\left(1-{p}_{1}\right)+{p}_{2}\left(1-{p}_{2}\right)\right]}{{\left({p}_{2}-{p}_{1}\right)}^{2}}$$

where:

$f$ denotes the cluster effect,

$\alpha$ denotes the first-species error,

$1-\beta$ denotes the statistical power.

Under these conditions, the required minimum was estimated at 19 health facilities for Ziniaré and Manga, and 16 for Ténado and Sapouy, accounting for a 20% expected non-response rate. In practice, the final allocation comprised 20 health facilities in Ziniaré, 20 in Manga, 16 in Ténado, and 16 in Sapouy, for a total of 72 health facilities.To ensure representativeness, Moran’s spatial autocorrelation was applied to classify facilities within each district into four spatial–performance clusters: high–high (HH), high–low (HL), low–high (LH), and low–low (LL), based on the average annual number of consultations for children under five from 2020 to 2022 [29]. This method allowed the sampling to capture heterogeneity in performance while maintaining statistical representativeness.

Within the selected facilities, health professionals were recruited using a non-probability convenience sampling approach proposed by MEASURE Evaluation [28]. This choice was necessitated by the prevailing security constraints in the study districts, which precluded a strictly randomised recruitment process. The number of respondents per facility was proportional to staff size and patient volume: in facilities with four providers or fewer, all eligible staff were surveyed, whereas in larger facilities three to four providers were purposively selected for interview. Ultimately, the study reached a total of 799 unique individual participants, comprising 518 facility-based professionals and 281 community-based health workers (CBHWs). It is important to clarify that because the MDE is an integrated suite of eight applications, its design assigns multiple tools to the same professional depending on their role. For example, a single facility head is responsible for clinical, logistical, and financial tools simultaneously. Consequently, these 799 individuals represent 1,136 cumulative “profile instances” (or user-tool observations) across the various functional domains analysed.

### Data collection

The MDE evaluation was designed as a longitudinal study comprising five survey waves scheduled between December 2023 and April 2026. The present research draws on the second wave, conducted in June 2024 across the four study districts, six months after the baseline survey. Data were collected through a structured questionnaire that captured healthcare providers’ socio-demographic profiles, their training experiences (both formal and informal), perceived ease of use, attitudes toward digital tools, and self-reported levels of mastery. Organisational characteristics and the operational capacities of participating facilities were also documented. The questionnaire was programmed in ODK Collect (version v2024.3.3); then 38 fieldworkers were trained for one week to use it for data collection across the 72 selected health facilities. The survey was administered face-to-face to healthcare providers and community-based health workers. Following collection, a rigorous data management process was undertaken, including checks for completeness, correction of inconsistencies, treatment of outliers, and verification of internal coherence and comparability with baseline data.

### Data analysis

The analysis sought to identify the determinants of healthcare providers’ uptake of the various components of the MDE. To account for heterogeneity in use, separate models were developed for six functional domains: service delivery (*REC-PCIME* and *REC-Maternité*), community health management (*E-SantéCom*), pharmaceutical supply management (*NetSIGL 2.0*), quality of care management (*E-Qualité*), financial flow management (*E-Flux financier*), and free healthcare management (*E-Gratuité* and *FIS*). User profiles were defined according to the design specifications of the MDE, which attribute each application to specific cadres of health personnel (e.g. all healthcare providers for clinical registers, community-based workers for *E-SantéCom*, pharmacists and facility managers for *NetSIGL 2.0*, and facility managers and treasurers for E-Flux financier). *REC-PCIME* and *REC-Maternité* tools were grouped under “service delivery” to reflect the operational reality of Burkina Faso’s primary health centres, where the same providers typically manage both pediatric and maternal care. Statistically, this aggregation ensures adequate sample sizes and model stability, while aligning with the MDE’s strategic objective of promoting an integrated approach to clinical care. Similarly, *E-Gratuité* and the *FIS* were grouped under **“**free healthcare management” primarily because they serve as the integrated operational pillars of Burkina Faso’s national free healthcare policy. The FIS captures the clinical proof of care, while E-Gratuité processes the financial claim. Their sequential use by the same provider in a single workflow also ensures statistical robustness and prevents data fragmentation in the models. For analytical purposes, the dependent variables captured actual use of each tool, measured as binary outcomes (1 = use, 0 = non-use), based on the self-reported declaration of each professional as to whether they used the MDE tools in their routine practice.

Independent variables were grouped into three domains: (i) sociodemographic characteristics (age, sex, education, and professional experience); (ii) organisational factors (type of facility, electricity supply, availability of internet); and (iii) contextual factors (exposure to formal training, peer support, perceived ease of use, self-assessed proficiency, and overall attitude toward the tools). Perceived ease of use was initially captured using a four-point Likert scale (ranging from ‘Very Difficult’ to ‘Very Easy’). To meet the requirements of multivariate logistic regression and ensure statistical clarity, this variable was dichotomised: responses of ‘Easy’ and ‘Very Easy’ were grouped into the ‘Easy’ category, while ‘Difficult’ and ‘Very Difficult’ were merged into the ‘Difficult’ category. This categorization allows for a direct assessment of how a favourable perception of tool usability is associated with its actual use (1 = use, 0 = non-use).

The analytical sequence combined descriptive, univariate, and multivariate stages. Descriptive statistics profiled respondents and patterns of tool use by district and facility. Univariate logistic regressions were then conducted for each tool, with variables retained for multivariate analysis if *p* < 0.20. Finally, multivariate logistic regression was used to estimate adjusted effects while controlling for confounders. The model specification was:

$$\begin{aligned} logit\left(P=1|X\right)&=ln\left(\frac{P\left(Y=1\right)}{P\left(Y=0\right)}\right)\cr&={\beta}_{0}+{\beta}_{1}{X}_{i1}+\cdots+{\beta}_{p}{X}_{ip}\end{aligned}$$

Where $P\left(Y=1\right)$ denotes the probability that a tool is used, $P\left(Y=0\right)$ the probability of non-use, ${\beta}_{0}$ is the model intercept, and ${\beta}_{i}$represent the coefficients associated with the explanatory variables${X}_{i}$.

Multicollinearity between independent variables was systematically assessed for all six multivariate models using the variance inflation factor (VIF). All VIF values remained within acceptable limits (typically < 5), confirming the absence of problematic redundancy between predictors such as education and professional experience. Detailed VIF results are provided in Supplementary Table 1.

Descriptive statistics and chi-square tests were performed using R (version 4.5.2). To ensure the robustness of the estimates against small sample sizes, rare events, and issues of complete or quasi-complete separation, univariate and multivariate logistic regressions were conducted using Firth’s penalized likelihood approach (firthlogit) in STATA (version 18). This method reduces bias in parameter estimates and provides better-calibrated 95% confidence intervals (CIs) compared to traditional maximum likelihood estimation in the presence of sparse data. Adjusted odds ratios (aOR) were calculated for each covariate, with statistical significance set at 5% in multivariate logistic models. 

## Results

### Sociodemographic characteristics of MDE tool users

Table 1 presents the sociodemographic characteristics of MDE tool users across district pairs (Ziniaré vs. Manga and Ténado vs. Sapouy). A statistically significant difference was observed for the age group variable between Ténado and Sapouy (*p* = 0.031). No significant differences were found for other sociodemographic characteristics between the compared districts.

**Table 1:** Sociodemographic characteristics of MDE tool users

Characteristics	Categories	Ziniaré	Manga	*p*-value^1^	Ténado	Sapouy	*p*-value^1^
Sex	Male	82 (36.3)	98 (43.9)	0.118	98 (53.3)	97 (58.4)	0.387
	Female	144 (63.7)	125 (56.1)		86 (46.7)	69 (41.6)	
Age group	< 30 years	22 (9.8)	27 (12.1)	0.233	9 (4.9)	16 (9.6)	0.031
	30–50 years	177 (78.7)	180 (80.7)		144 (78.3)	135 (81.3)	
	> 50 years	26 (11.6)	16 (7.2)		31 (16.8)	15 (9)	
Education	Primary	49 (21.7)	32 (14.3)	0.302	26 (14.1)	28 (16.9)	0.674
	Secondary	144 (63.7)	156 (70)		126 (68.5)	108 (65.1)	
	University	27 (11.9)	31 (13.9)		31 (16.8)	27 (16.3)	
	None	3 (1.3)	2 (0.9)			2 (1.2)	
	Other	3 (1.3)	2 (0.9)		1 (0.5)	1 (0.6)	
Professional role	Facility head	20 (8.8)	20 (9)	0.996	16 (8.7)	16 (9.6)	0.243
	Health worker	83 (36.7)	82 (36.8)		80 (43.5)	55 (33.1)	
	DMEG Manager	21 (9.3)	24 (10.8)		16 (8.7)	17 (10.2)	
	Manager	2 (0.9)	1 (0.4)		1 (0.5)	1 (0.6)	
	Cashier	2 (0.9)	2 (0.9)			1 (0.6)	
	COGES Treasurer	18 (8)	16 (7.2)		15 (8.2)	9 (5.4)	
	CBHW	80 (35.4)	78 (35)		56 (30.4)	67 (40.4)	
Literacy (CBHW)	Literate	76 (95)	74 (94.9)	> 0.9	56 (100)	64 (95.5)	0.249
	Illiterate	4 (5)	4 (5.1)			3 (4.5)	
Distance to facility (CBHW)	< 5 km	40 (50)	44 (56.4)	0.517	31 (55.4)	39 (58.2)	0.892
	> 5 Km	40 (50)	34 (43.6)		25 (44.6)	28 (41.8)	

^1^chi² test or Fisher’s exact test

### Proportion of users by type of use and by district

Table 2 highlights significant disparities in the use of digital tools across districts for various application types. Ziniaré stands out for its high adoption rates in several categories, particularly in service delivery (86.4%, *p* < 0.001) and financial flow management (96.2%, *p* < 0.001). In contrast, Manga shows very low adoption in these areas, with only 5.4% for service delivery and no usage at all for financial flow management.

In terms of pharmaceutical supply management, Ténado reports a very high adoption rate (89.9%, *p* = 0.001), while Sapouy follows with a more moderate rate (70.6%). Significant differences are also observed in quality of care management, with 64.4% application usage in Ténado, compared to no usage in Sapouy (*p* < 0.001).

For free care management, adoption rates are more homogeneous across districts, with 49.6% in Ziniaré and 46.4% in Sapouy, showing no statistically significant difference (*p* = 0.692).

Finally, in community health management, the differences are not statistically significant. Usage rates are very high in Ziniaré (97.5%) and Manga (98.7%), while there is no usage reported in either Ténado or Sapouy.

**Table 2:** Proportion of users by type of digital health application and by district

Digital health application type		Ziniaré		Manga		*p*-value^1^	Ténado		Sapouy		*p*-value^1^
		*N*	*n*(%)	*N*	*n*(%)		*N*	*n*(%)	*N*	*n*(%)	
Service delivery (*REC-PCIME* & *REC-Maternité*)		280	242 (86.4)	148	8 (5.4)	< 0.001	248	205 (82.7)	179	139 (77.7)	0.243
Community health management (*E-SantéCom*)		80	78 (97.5)	78	77 (98.7)	> 0.9	56	0 (0%)	67	0 (0%)	0.321
Pharmaceutical supply management (*NetSIGL 2.0*)		131	27 (20.6)	47	12 (25.5)	0.621	109	98 (89.9)	85	60 (70.6)	0.001
Quality of care management (*E-Qualité*)		112	75 (67)	23	0 (0%)	< 0.001	87	56 (64.4)	61	0 (0%)	< 0.001
Financial flow management (*E-Flux financier*)		130	125 (96.2)	39	0 (0%)	< 0.001	102	97 (95.1)	70	11 (15.7)	< 0.001
Free healthcare management (*E-Gratuité* and *FIS*)		280	139 (49.6)	148	39 (26.4)	< 0.001	248	121 (48.8)	179	83 (46.4)	0.692

^1^chi² test or Fisher’s exact test

### Univariate logistic regression models

Table 3 presents the results of the univariate analysis and identifies variables to be included in the multivariate logistic regression models (*p* < 0.2). The univariate analysis revealed several significant factors across different models: sex, age, professional experience, education level, perceived ease of use, tool proficiency, and district were found to influence application usage. These factors varied across models. For instance, gender and education played key roles in service delivery and pharmaceutical supply management, whereas perceived ease of use was a critical determinant for community health management. The results of this step were subsequently incorporated into multivariate logistic regression models to refine the analysis

**Table 3:** Univariate regression model

Factors	Modalities compared (Reference)	Service delivery	Community health management	Pharmaceutical supply management	Quality of care management	Financial flow management	Free healthcare management
		OR (*p*-value)	OR (*p*-value)	OR (*p*-value)	OR (*p*-value)	OR (*p*-value)	OR (*p*-value)
Sex	Male (Female)	1.5 (**0.003**)	0.5 (**0.004**)	2.7 **(< 0.001**)	0.4 (**0.011**)	1.1 (0.741)	4.7 **(< 0.001**)
Age Group	30–50 years (< 30 years)	7.1 **(< 0.001**)	0.5 (**0.105**)	2.1 (**0.118**)	-	3.9 (**0.090**)	1.1 (0.869)
	> 50 years (< 30 years)	6.1 **(< 0.001**)	0.2 (**0.012**)	2.6 (**0.126**)	2.3 (**0.019**)	4.4 (**0.077**)	1.2 (0.564)
Education	Secondaire (Primaire)	31.6 **(< 0.001**)	0.7 (0.280)	5.8 (**0.012**)	-	2.7 (**0.028**)	1.5 (0.267)
	Universitaire (Primaire)	75.3 **(< 0.001**)	NA	6.3 (**0.010**)	1.1 (0.705)	1.7 (0.242)	1.9 (**0.087**)
Professional experience	5–10 years (< 5 years)	2.2 **(< 0.001**)	1.1 (0.656)	1.8 (0.211)	0.6 (0.460)	3.5 (**0.004**)	3.5 **(< 0.001**)
	> 10 years (< years)	5.2 **(< 0.001**)	0.7 (0.426)	1.7 (**0.188**)	0.7 (0.611)	4.0 **(< 0.001**)	7.4 **(< 0.001**)
Literacy	Literate (Illeterate)	NA	0.5 (0.272)	NA	NA	NA	NA
Distance to health facility	Less than 5 Km (More than 5 Km)	NA	0.8 (0.344)	NA	NA	NA	NA
Level of care	CM (CSPS)	0.5 (**0.004**)	0.7 (0.312)	2.3 (**0.051**)	0.6 (0.373)	0.7 (0.397)	0.5 (**0.002**)
District	Manga (Ziniaré)	0.0 (< **0.001**)	1.6 (0.634)	1.3 (0.458)	0.0 (**0.002**)	0.0 **(< 0.001**)	0.4 **(< 0.001**)
	Ténado (Ziniaré)	0.7 (0.232)	0.0 **(< 0.001**)	32.5 **(< 0.001**)	0.9 (0.699)	0.8 (0.683)	1.0 (0.845)
	Sapouy (Ziniaré)	0.5 (**0.015**)	0.0 **(< 0.001**)	9.0 **(< 0.001**)	0.0 **(< 0.001**)	0.0 **(< 0.001**)	0.9 (0.495)
Energy availability	Yes (No)	1.3 (0.426)	0.7 (0.428)	1.2 (0.611)	1.6 (0.373)	3.9 (**0.004**)	0.4 (**0.015**)
Internet availability	Yes (No)	1.3 (0.206)	1.4 (0.275)	1.1 (0.608)	0.9 (0.675)	2.7 (**0.001**)	1.0 (0.975)
Tablets availability	More than 4 tablets (4 tablets or less)	3.1 **(< 0.001**)	0.6 (**0.061**)	0.6 (**0.076**)	1.0 (0.921)	1.5 (**0.104**)	1.1 (0.530)
Formal training	Yes (No)	0.1 (**0.149**)	NA	NA	NA	NA	30.8 (**0.018**)
Peer training	Yes (No)	0.6 (0.378)	NA	1.6 (0.645)	1.6 (0.661)	0.7 (0.836)	0.2 (**0.005**)
Ease of use	Easy (Difficult)	8.3 **(< 0.001**)	2468.9 **(< 0.001**)	0.7 (0.239)	0.8 (0.472)	3.8 (**< 0.001**)	2.9 **(< 0.001**)
Mastery of the tool	Mastery (Non-mastery)	4.3 (**0.010**)	NA	5.3 (0.261)	0.4 (0.376)	6.0 (0.297)	3.1 (**0.011**)
Attitude towards the tool	Positive (negative)	1.2 (0.901)	NA	2.3 (0.579)	0.3 (0.399)	5.0 (0.316)	0.3 (0.241)

Note: NA : Not applicable. This indicates that the variable was not collected for the specific professional category (cadre) associated with that tool’s functional domain, as the questionnaire was modular and targeted to specific user roles

### Multivariate logistic analysis

#### Factors associated with the use of service delivery tools

The multivariate analysis shows in Table 4 that the use of service delivery tools is strongly associated with education level and professional experience. Healthcare professionals with secondary (OR = 3.475) or university-level education (OR = 88.487), as well as those with more than 10 years of professional experience (OR = 245.695), are significantly more likely to use these tools. Diagnostic tests for this model confirmed no significant collinearity between education level (VIF = 0.17) and professional experience (VIF = 1.18) (see Supplementary Table 1). The high adjusted Odds Ratio (aOR = 245.69) observed for experience over 10 years is therefore not an artifact of multicollinearity but reflects a strong association in the data, albeit with a wide confidence interval due to sparse data issues in the reference categories.

**Table 4:** Factors associated with the use of care delivery tools (REC-PCIME and REC-Maternité), N = 855

*Factors*	*Modalities*	*N*	*n (%)*	*OR*	*CI 95%*
Sex	Female	390	251 (64.4)	–	–
	Male	465	343 (73.8)	0.048	0.002–1.283
Age group	< 30 years	42	11 (26.2)	–	–
	30–50 years	702	507 (72.2)	0.056	0.000–10.737
	> 50 years	110	76 (69.1)	0.003	0.000–1.488
Education	Primary school	38	2 (5.3)	–	–
	Secondary school	583	399 (68.4)	**3.475**	**1.678–7.197**
	University	230	193 (83.9)	**88.487**	**45.318–1 728.167**
Professional experience	< 5 years	125	54 (43.2)	–	–
	5–10 years	245	153 (62.5)	2.177	0.118–40.220
	> 10 years	485	387 (79.8)	**245.695**	**2.552 – 23 650.623**
Tablets availability	4 tablets or less than	233	119 (51.1)	–	–
	More than 4 tablets	622	475 (76.4)	0.814	0.040–16.496
Formal training	No	20	20 (100.0)	–	–
	Yes	98	82 (83.7)	0.274	0.003–29.540
Ease of use	Difficult	231	82 (35.5)	–	–
	Easy	624	512 (82.1)	6.446	0.214–193.997
Mastery of the tool	Non-mastery	27	22 (81.5)	-	-
	Mastery	212	201 (94.8)	6.443	0.080–517.012

Note: The discrepancies between the sum of sample sizes and the total (N) are due to rare missing data and the use of a modular questionnaire, where certain variables were collected only from the relevant professional categories

#### Factors associated with the use of the community health tool (E-SantéCom)

Table 5 presents the results of the multivariate analysis for the use of the *E-SantéCom* tool, revealing that perceived ease of use is a strong predictor of adoption. Users who perceive the tool as easy to use are significantly more likely to use it (OR = 98.512), highlighting its critical importance.

Regarding geographic distribution, the districts of Ténado (OR = 0.009) and Sapouy (OR = 0.005) show an almost complete absence of application usage, in stark contrast to Ziniaré and Manga, where use is nearly universal. The extremely wide confidence interval for the “Easy” category (95% CI: 1.77–5492.15) is due to a “sparse data” issue and quasi-perfect separation in the logistic regression model. Specifically, 100% (*n* = 141) of community workers who perceived the tool as easy to use actually used it, compared to only 10% (*n* = 14) in the “Difficult” category. While this distribution results in a very high Odds Ratio and a wide interval, the model’s stability was confirmed by a low Variance Inflation Factor (VIF = 0.42), ruling out multicollinearity.

**Table 5:** Factors associated with use of the community health tool (E-SantéCom), *N* = 281

Factors	Modalities	*N*	*n* (%)	OR	CI 95%
Sexe	Female	136	87 (64.0)	–	–
	Male	145	68 (46.9)	0.795	0.067–9.443
Age group	< 30 years	39	27 (69.2)	–	–
	30–50 years	217	119 (54.8)	3.633	0.038–343.517
	> 50 years	25	9 (36.0)	0.943	0.006–139.703
District	Ziniaré	80	78 (97.5)	–	–
	Manga	78	77 (98.7)	10.203	0.429–242.637
	Ténado	56	0 (0.0)	**0.009**	**0.000–0.215**
	Sapouy	67	0 (0.0)	**0.005**	**0.000–0.135**
Tablets availability	4 tablets or less than	90	57 (63.3)	–	–
	More than 4 tablets	191	98 (51.3)	6.970	0.220–220.882
Ease of use	Difficult	140	14 (10.0)	–	–
	Easy	141	141 (100.0)	**98.512**	**1.767–5 492.151**

#### Factors associated with the use of the pharmaceutical management tool (NetSIGL 2.0)

The multivariate analysis of the use of the NetSIGL 2.0 tool for pharmaceutical management reveals several significant findings. Gender plays an important role, with men being three times more likely to use the tool than women (OR = 3.072). Education level also has a significant association: professionals with secondary (OR = 5.443) or university-level education (OR = 4.509) are considerably more likely to use the tool compared to those with only primary education. Finally, tablet availability also influences usage, with lower adoption among those with more than four tablets available (OR = 0.562) (see Table 6).

**Table 6:** Factors Associated with the use of the pharmaceutical management tool (NetSIGL 2.0), *N* = 372

Factors	Modalities	*N*	*n* (%)	OR	CI 95%
Sex	Female	92	32 (34.8)	–	–
	Male	280	165 (58.9)	**3.072**	**1.762–5.357**
Education	Primary school	14	2 (14.3)	–	–
	Secondary school	249	134 (53.8)	**5.443**	**1.326–22.338**
	University	109	61 (56.0)	**4.509**	**1.061–19.160**
Professional experience	< 5 years	23	9 (39.1)	–	–
	5–10 years	83	45 (54.2)	1.955	0.730–5.230
	> 10 years	266	143 (53.8)	1.329	0.530–3.332
Tablets availability	4 tablets or less than	108	65 (60.2)	–	–
	More than 4 tablets	264	132 (50.0)	**0.562**	**0.348–0.906**

#### Factors associated with the use of the quality of care management tool (E-Qualité)

The multivariate analysis of the use of the *E-Qualité* tool reveals significant geographic disparities. In Manga and Sapouy, application usage is virtually nonexistent (OR = 0.011 and 0.004, respectively), which may be explained by the staggered deployment of MDE tools. In contrast, usage is high in Ziniaré (67%) and Ténado (64.4%), reflecting more advanced stages of implementation. While healthcare professionals over 50 years old show a favorable trend (OR = 2.291), and women appear to adopt the tool more readily than men, these associations are not statistically significant (see Table 7).

**Table 7:** Factors associated with the use of the quality of care management tool (E-Qualité), *N* = 283

Factors	Modalities	*N*	*n* (%)	OR	CI 95%
Sex	Female	36	24 (66.7)	–	–
	Male	247	107 (43.3)	0.398	0.149–1.060
Age group	30–50 years	244	106 (43.4)	–	–
	> 50 years	39	25 (64.1)	2.291	0.906–5.791
District	Ziniaré	112	75 (67.0)	–	–
	Manga	23	0 (0.0)	**0.011**	**0.001–0.182**
	Ténado	87	56 (64.4)	0.839	0.460–1.529
	Sapouy	61	0 (0.0)	**0.004**	**0.000–0.069**

#### Factors associated with the use of the financial flow management tool (E-Flux financier)

The multivariate analysis indicates that the use of the *E-Flux financier* tool is strongly associated with education level, with significantly higher usage among professionals with secondary education (OR = 7.658). Geographic disparities are also prominent: usage is nearly nonexistent in Manga and Sapouy, whereas in Ziniaré and Ténado, the tool is used almost universally. Although professional experience and perceived ease of use show a positive trend, these associations are not statistically significant in this model (see Table 8).

**Table 8:** Factors associated with the use of the financial flow management tool (E-Flux financier), *N* = 341

Factors	Modalities	*N*	*n* (%)	OR	CI 95%
Age group	< 30 years	6	2 (33.3)	–	–
	30–50 years	278	190 (68.4)	0.280	0.004–22.213
	> 50 years	56	40 (71.4)	0.189	0.002–20.031
Education	Primary school	22	11 (50.0)	–	–
	Secondary school	206	150 (72.8)	**7.658**	**1.452–40.375**
	University	109	69 (63.3)	1.879	0.312–11.297
Professional experience	< 5 years	38	15 (39.5)	–	–
	5–10 years	57	40 (70.2)	3.050	0.402–23.154
	> 10 years	246	178 (72.4)	3.320	0.551–19.987
District	Ziniaré	130	125 (96.2)	–	–
	Manga	39	0 (0.0)	**0.001**	**0.000–0.014**
	Ténado	102	97 (95.1)	**0.940**	**0.233–3.796**
	Sapouy	70	11 (15.7)	**0.008**	**0.002–0.026**
Energy availability	No	19	7 (36.8)	–	–
	Yes	322	226 (70.2)	1.423	0.232–8.745
Internet availability	No	57	28 (49.1)	–	–
	Yes	284	205 (72.2)	2.371	0.747–7.525
Tablets availability	4 tablets or less than	97	60 (61.9)	–	–
	More than 4 tablets	244	173 (70.9)	1.504	0.459–4.926
Ease of use	Difficult	82	37 (45.1)	–	–
	Easy	259	196 (75.7)	3.146	0.922–10.737

Note: The discrepancies between the sum of sample sizes and the total (N) are due to rare missing data and the use of a modular questionnaire, where certain variables were collected only from the relevant professional categories

#### Factors associated with the use of free care management tools (E-Gratuité and FIS)

Table 9 shows that the use of *E-Gratuité* tools is strongly associated with gender, with men being significantly more likely to use the tools (OR = 18.044), and by professional experience, with higher usage among professionals with more than 10 years of experience (OR = 12.052).

**Table 9:** Factors associated with the use of free care management tools (E-Gratuité and FIS), *N* = 855

Factors	Modalities	*N*	*n* (%)	OR	CI 95%
Sex	Female	390	98 (25.1)	–	–
	Male	465	284 (61.1)	**18.044**	**5.144–63.297**
Education	Primary school	38	13 (34.2)	–	–
	Secondary school	583	255 (43.7)	21.117	0.820–544.099
	University	230	114 (49.6)	21.335	0.767–593.687
Professional experience	< 5 years	125	18 (14.4)	–	–
	5–10 years	245	92 (37.6)	8.907	0.849–93.489
	> 10 years	485	272 (56.1)	**12.052**	**1.588–91.457**
Energy availability	No	39	25 (64.1)	–	–
	Yes	816	357 (43.8)	0.061	0.003–1.196
Formal training	No	20	0 (0.0)	–	–
	Yes	98	42 (42.9)	14.530	0.647–326.436
Peer training	No	84	37 (44.1)	–	–
	Yes	34	5 (14.7)	1.649	0.386–7.038
Ease of use	Difficult	231	61 (26.4)	–	–
	Easy	624	321 (51.4)	1.427	0.095–21.360
Mastery of the tool	Non-mastery	27	7 (25.9)	–	–
	Mastery	212	113 (53.3)	14.843	0.638–345.124

Note: The discrepancies between the sum of sample sizes and the total (N) are due to rare missing data and the use of a modular questionnaire, where certain variables were collected only from the relevant professional categories

## Discussion

This study examined the factors of digital tools use within the MDE in Burkina Faso and revealed significant results according to tool type, sociodemographic features of users, and healthcare facility context. By confronting these results with existing literature, both from Sub-Saharan Africa and broader global health research, the discussion highlights the interplay of individual, organisational, and systemic factors linked to health professionals’ engagement with digital technologies.

A central finding of this study is the role of education as a factor for the use of service delivery and financial management tools. Healthcare professionals with secondary or university-level education were much more likely to adopt the service delivery tools (*REC-PCIME* and *REC-Maternité*) and *E-Flux financier* tool. This finding confirms what has been widely reported in digital health research: higher education increases digital literacy and enhances the ability to integrate technology into clinical workflows [30]. Studies in other low- and middle-income countries (LMICS) have similarly shown that more educated professionals engage more readily with electronic record-keeping and financial systems, while those with fewer qualifications face exclusionary barriers [31]. Such disparities underline a persistent equity concern. Unless targeted training strategies are implemented, healthcare workers with lower educational backgrounds risk being systematically disadvantaged by digital transformation and, ultimately, patients in facilities where these workers predominate may also receive lower-quality care.

The importance of perceived ease of use emerged particularly in the adoption of the *E-SantéCom* platform for community health management. This is consistent with the UTAUT strategy [21], which identifies effort expectancy as a critical predictor of technology use. In our context, *E-SantéCom* users are community-based health workers recruited from rural areas with little formal education, making intuitive design a precondition for use. Similar evidence from Rwanda demonstrated that technologies with simplified interfaces and embedded decision support are more easily embraced by front-line health workers with limited digital exposure [25]. These findings emphasize that technological design is not a purely technical matter but a determinant of health equity. Human-centered design, usability testing, and participatory approaches in tool development are essential for broad-based use, especially when users are drawn from disadvantaged areas as rural environments.

Gender differences also played a crucial role in shaping use. Men were far more likely than women to use the pharmaceutical management tool *NetSIGL 2.0*, reflecting broader gender divides in the use of health digital tools. Scholars have long noted that women in LMICs face structural and sociocultural constraints to their participation in digital innovation, including unequal access to training, restrictive gender norms, and double workloads linked to professional and domestic responsibilities [24]. Except for age Ténado-Sapouy, there are no district variations by sex or education (Table 1, *p* > 0.05). Higher *NetSIGL 2.0* (aOR 3.07, Table 6) and *E-Gratuité* use (aOR 18.04, Table 9) may be explained by men in management positions (facility heads/DMEG). Indeed, our data provide strong evidence for this concentration, as 87.3% (247 out of 283) of facility heads in the study districts were men. In our study, this disparity also intersects with institutional practices, as managerial tools defined specifically as applications for pharmaceutical logistics (*NetSIGL 2.0*), financial oversight (*E-Flux financier*), and policy management (*E-Gratuité/FIS*) are more often allocated to male staff. This suggests that the “gender gap” in tool use is primarily a reflection of structural inequities in the distribution of professional roles rather than a difference in individual interest. Similarly, professional experience, especially above ten years, emerged as a facilitator of digital use in the context of free care management systems such as *E-Gratuité* and *FIS*. Experienced health professionals may possess greater confidence in administrative routines and a deeper knowledge of patient management processes, which in turn eases their engagement with digital versions of routine documentation. These findings are coherent with prior research emphasising that professional longevity can promote digital use, although it may also interact with pre-existing hierarchies that raise barriers for younger or less-experienced staff [32].

Geographical and infrastructural disparities reinforce inequalities in the use of digital tools. Our study shows marked contrasts in the use of *E-Qualité* for managing the quality of care, with entire districts reporting very marginal use. The low use of *E-Qualité* in Manga/Sapouy could be explained by the limited deployment of MDE and the limited coverage of the World Bank’s PRSS (Health Services Strengthening Project), which focused on quality assessment mainly in the Ziniaré/Ténado regions. This mirrors findings from sub-Saharan Africa, where unreliable electricity supplies and limited internet connectivity create deep spatial inequalities in the implementation of digital health [33]. Infrastructure inequality means that rural facilities face greater challenges, not only because of weaker networks, but also because staff in these areas often have fewer opportunities for training and support.

An unexpected observation was that facilities with a greater number of devices, particularly more than four tablets, were less likely to adopt certain tools, in this case *NetSIGL 2.0* (aOR 0.56; 95% CI: 0.35–0.91), which is primarily used by DMEG. While this counter-intuitive result might suggest a diffusion of responsibility or technological overload, a more nuanced explanation lies in the professional distribution of tasks within larger facilities. In health facilities with more equipment which are typically larger professional roles are often more specialized. In these settings, the use of pharmaceutical management tools is strictly delegated to the designated DMEG, whereas in smaller facilities with fewer tablets, staff polyvalence may lead more providers to engage with the tool. This suggests that lower individual usage in larger facilities reflects professional specialization rather than a failure of use. Nevertheless, this result also highlights the risks of inadequate preparation on how to integrate multiple platforms into institutions’ workflows and confirms that making technical resources available without thorough training and supervision is insufficient for sustainable use. Further comprehensive and longitudinal studies are required to fully elucidate the organisational mechanisms driving this counter-intuitive dynamic.

Beyond individual and institutional factors, technological challenges such as interoperability continue to constrain the effectiveness of digital ecosystems. As noted by Basajja et al. (2022) in Uganda [5], the coexistence of multiple, non-interoperable tools generates fragmented information systems and diminishes the potential of digital health to improve efficiency. Our findings reflect this issue indirectly, as healthcare workers reported reluctance to engage with some tools that operated in isolation or required repeated data entry. The governance of health information systems must therefore prioritize interoperability, standardized data flows, and secure information management [14], both to ease use by frontline staff and to build institutional trust in digital solutions.

When interpreted in light of previous research, the results of this study confirm that digital health use in low-resource contexts is rarely constrained by access to technology alone. Rather, it depends on the conjunction of education, professional experience, ease of use, equitable gender dynamics, and robust infrastructural support. This confirms the broader insights of scholars who stress that organisational culture, managerial leadership, and institutional support are decisive in determining whether digital health interventions succeed or stagnate [21, 24]. At the same time, our findings add nuance by demonstrating the particular sensitivity of community-based tools to usability design, highlighting that simplicity itself is a factor of equity in digital health access.

### Public health implications

These results have several significant implications for public health and require concrete, context-specific actions. First, to bridge the digital literacy gap, the Ministry of Health should move beyond generic workshops toward decentralised peer-mentoring schemes. Since healthcare professionals with over 10 years of experience were identified as high adopters for certain tools, they could serve as “digital champions” to provide on-site coaching to less experienced colleagues within their respective CSPS.

Second, for CBHWs who often have limited formal education, the *E-SantéCom* application must move toward multilingual audio-visual training modules (e.g., in Mooré, Dioula, or Fulfuldé) integrated directly into the tablets. This human-centered design ensures that literacy is not a barrier to digital health equity in rural areas.

Third, addressing gender inequities requires proactive institutional policies. Since men are currently more likely to use managerial tools like *NetSIGL 2.0*, we recommend targeted training quotas for female providers to access specialised roles such as Medicine Depot Manager. Dedicated mentorship programs for women could help break structural barriers and ensure equitable access to all digital management platforms.

Fourth, regarding infrastructure, investments must be prioritised for rural districts like Ténado and Sapouy, where connectivity is weakest. Specifically, the deployment of solar-powered charging kits for tablets is an essential pragmatic solution to ensure tool availability despite unstable electricity grids.

Finally, as suggested by the fragmentation of the digital landscape, the Ministry of Health must strengthen digital governance by adopting a clear enterprise architecture. This involves prioritizing interoperability between the MDE and the ENDOS-BF to reduce redundant data entry and embed these tools into everyday clinical and administrative practice.

### Limitations and future considerations

Despite its contributions, this study has certain limitations. The cross-sectional design prevents the inference of causal relationships, and the use of self-reported data introduces potential biases, including a social desirability bias. Furthermore, the use of non-probability convenience sampling may introduce a potential selection bias and limits the generalisability of the findings. This is particularly pertinent given that the study focuses on only four health districts out of the 70 nationwide, which may restrict the representativeness of the results for Burkina Faso as a whole. It is also important to note that, although 1,136 “profile instances” were analysed, these stem from 799 unique individual participants; the fact that certain professionals, particularly those in leadership positions, are designated users for multiple tools may influence the weighting of specific profiles in the overall findings. Future research should use longitudinal approaches and mixed methods to capture both changing patterns of tool use and the lived experiences of health professionals. Incorporating qualitative perspectives could shed light on how socio-cultural norms, gender roles and institutional cultures influence the use of digital health. In addition, more attention needs to be paid to the long-term sustainability of digital health ecosystems, particularly in terms of interoperability between platforms, data governance, privacy protection and the integration of dashboards into overall health management by public decision-makers.

## Conclusion

This study identified education level, perceived ease of use, gender, professional experience, and infrastructural factors as key factors of digital tool use within the MDE in Burkina Faso. Education consistently predicted use of service delivery and financial management tools, while ease of use proved critical for community-based applications. Gender and experience influenced managerial tools, revealing persistent inequities in access to digital health. Geographic disparities driven by deficiencies in electricity and internet connectivity further constrained adoption in rural districts.

These findings underscore that digital transformation in health relies not only on technology provision but also on addressing entrenched structural barriers. Targeted training adapted to varying educational levels, gender-responsive capacity-building, investments in infrastructure, and human-centred design are essential to achieve equitable and effective use of digital health tools. By integrating these measures, health systems in Burkina Faso and comparable low-resource contexts can unlock the full potential of digital ecosystems to strengthen equity, efficiency, and the overall quality of care.

## Supplementary Information

Below is the link to the electronic supplementary material.

Supplementary Material 1

## Data Availability

The datasets generated and/or analyzed during the current study are not publicly available but may be made available upon reasonable request. Access will be granted to qualified researchers who submit a formal proposal and demonstrate a legitimate interest in the data. Requests should be addressed to the corresponding author.

## Abbreviations

MDE: Minimal Digital Ecosystem
REC-PCIME: Electronic Consultation Register for Integrated Management of Childhood Illness
REC-Maternité: Electronic Consultation Register for Maternal Care
E-SantéCom: Community Health Digital Platform
NetSIGL 2.0: Pharmaceutical Supply Management System
E-Qualité: Quality of Care Management Tool
E-Flux financier: Financial Flow Management Tool
E-Gratuité: Free Healthcare Management Tool
FIS: Individual Electronic Care Record
CSPS: Primary Health Care Facility
CM: Medical Center
CBHW: Community-Based Health Worker
COGES: Health Facility Management Committee
DMEG: Essential Medicines Depot
ENDOS-BF: National Health Data Warehouse
PRSS: Health Services Strengthening Project
DHIS2: District Health Information Software 2
aOR: adjusted Odds Ratio
CI: Confidence Interval
OR: Odds Ratio
LMICs: Low- and Middle-Income Countries
ODK: Open Data Kit
PHC: Primary Health Care
TAM: Technology Acceptance Model
UTAUT: Unified Theory of Acceptance and Use of Technology
