# Supplementary material for: Factors associated with digital tools use among primary healthcare professionals in Burkina Faso: a cross-sectional study of the minimal digital ecosystem
Source: BMC Health Serv Res. 2026 Mar 12;26:550. doi: 10.1186/s12913-026-14331-6 (PMC13094088; doi:10.1186/s12913-026-14331-6)
Supplement: Supplementary file 1 — Supplementary Material 1 [file 12913_2026_14331_MOESM1_ESM.pdf]

EDM-Q2 QUESTIONNAIRE

<table>
<tr><th>Revised specific objectives</th><th>Response procedures</th><th>Target professionals</th><th>Instructions</th></tr>
<tr><td>HEALTH DISTRICT CODE</td><td></td><td></td><td></td></tr>
<tr><td>HEALTH FACILITY CODE</td><td></td><td></td><td></td></tr>
<tr><td>Respondent profile</td><td></td><td></td><td></td></tr>
<tr><td>Q01 : How old are you?</td><td>|__|__| years</td><td></td><td></td></tr>
<tr><td>Q02 : What age group do you belong to? (If no answer regarding exact age)</td><td>Under 18 ☐/18-30 years ☐/31-40 years ☐/41-55 years ☐/56 years old and older ☐</td><td></td><td></td></tr>
<tr><td>Q03 : Gender of respondent</td><td>Male ☐/Female ☐</td><td></td><td></td></tr>
<tr><td>Q04 : What is your level of education?</td><td>None ☐/Primary school /Secondary school (First round) ☐/Secondary school (second round) ☐/High School ☐</td><td></td><td></td></tr>
<tr><td>Q05 : What is your marital status?</td><td>Single ☐/ In a relationship (monogamy) ☐/ In a relationship (polygamy) ☐/ Separated/Widowed ☐</td><td></td><td></td></tr>
<tr><td>Q06 : Where do you live? (town or neighborhood)</td><td>……………………………………………………………………….</td><td></td><td></td></tr>
<tr><td>Q07 : What is your professional category?</td><td>Physician ☐/ Pharmacist ☐/SFE/ME ☐/IDE/IB ☐/AA ☐/AB ☐/AIS (ASC) ☐/AV ☐/ / Hospital Administrator-Manager ☐/ Health attaché ☐/ ASSOG ☐/CSIO ☐ /Other☐(specify……………………………………………….)</td><td></td><td></td></tr>
<tr><td>Q08 : In what year did you first take up your position (recruitment into the civil service)?</td><td>|__|__|__|__|</td><td></td><td></td></tr>
<tr><td></td><td></td><td></td><td></td></tr>
<tr><td>Q09 : What is your profile?</td><td>CBHW ☐/ Cashier ☐/Manager DMEG ☐/ health worker ☐/ECD Member ☐/SISSE ☐/Manager ☐/ Facility manager ☐/ Implementing actor (STRFS/DCAPS/DSI/NGO) ☐/COGES Treasurer ☐</td><td></td><td></td></tr>
<tr><td>Q10 : What is your position of responsibility?</td><td>MCD ☐/DRD Manager ☐/ Facility manager ☐/PEV manager ☐/Maternity Manager ☐/SISSE ☐/MRACQS ☐/RAF ☐/ Regisseurs /None ☐/Other☐(specify……………………………………………….)</td><td></td><td></td></tr>
<tr><td>Q11 : q08 How long have you held this position of responsibility?</td><td>|__|__|__| month</td><td></td><td></td></tr>
<tr><td>Q12 : Since what year have you been working in this healthcare facility?</td><td></td><td></td><td></td></tr>
<tr><td>Q13 : What is your professional category (other than CBHW activity)?</td><td>Cultivator ☐/ Breeder ☐/ Fisherman ☐/ Merchant ☐/ Artisan ☐/ Spar ☐/ Other☐/</td><td></td><td></td></tr>
<tr><td>Q14 : Are you able to read and write in French (If yes, check Literate. If no, check Illiterate)?</td><td>Literate ☐/ illiterate ☐</td><td></td><td>CBHW</td></tr>
<tr><td>Q15 : How far is your home from the health facility?</td><td>Less than 5 km ☐/ More than 5 km ☐/</td><td></td><td></td></tr>
</table>

<table>
<tr><th colspan="4">Analyze the process of deploying MDE tools</th></tr>
<tr><td>Q16 : What is the total amount budgeted by your organization for the implementation of MDE activities (purchase of equipment, training of stakeholders, monitoring, supervision, coordination, etc.)?</td><td>## ### ### ###</td><td rowspan="2">STRFS/DCAPS/DSI/NGO</td><td></td></tr>
<tr><td>Q17 : What is the total amount actually mobilized by your organization for the implementation of MDE activities (purchase of equipment, training of stakeholders, monitoring, supervision, coordination, etc.)?</td><td>## ### ### ###</td><td></td></tr>
<tr><td>Q18 : How many functional tablets/phones do you have in your healthcare facility?</td><td>##</td><td rowspan="5">Facility manager</td><td></td></tr>
<tr><td>Q19 : How many tablets/phones are dedicated to MDE tools?</td><td>##</td><td></td></tr>
<tr><td>Q20: Do you consider the number of tablets/phones available in your Health facility (HF) sufficient for the implementation of MDE?</td><td>Yes/No</td><td></td></tr>
<tr><td>Q20_1 : If not, why not? (Give 2 to 3 reasons)</td><td>Text</td><td></td></tr>
<tr><td>Q20_2 : If not, how many consultation booths are you able to set up?</td><td>##</td><td></td></tr>
<tr><td>Q21 : What are the available staff numbers (by profile) in your HF?</td><td></td><td rowspan="13">Facility manager</td><td></td></tr>
<tr><td>State-registered nurses (IDE)</td><td>##</td><td></td></tr>
<tr><td>Registered nurses (IB)</td><td>##</td><td></td></tr>
<tr><td>Community health workers (AIS)</td><td>##</td><td></td></tr>
<tr><td>Midwife/Maieutician (SFE/ME)</td><td>##</td><td></td></tr>
<tr><td>Certified midwife (AB)</td><td>##</td><td></td></tr>
<tr><td>Auxiliary midwife (AA)</td><td>##</td><td></td></tr>
<tr><td>Manager of Essential Generic Medicines Depot (DMEG)</td><td>##</td><td></td></tr>
<tr><td>Pediatric health attaché</td><td>##</td><td></td></tr>
<tr><td>General physician</td><td>##</td><td></td></tr>
<tr><td>CBHW</td><td>##</td><td></td></tr>
<tr><td>Other (specify)</td><td>Text</td><td></td></tr>
<tr><td>Q22 : Are these numbers sufficient for the implementation of MDE?</td><td>Yes/No</td><td></td></tr>
</table>

Q18_1 : If not, give 2 or 3 reasons.	Text		
Q23 : When did you start using MDE tools in your HF ?			
E-Qualité	##/#### month/year		
E-Flux financier	##/#### month/year		
REC-Maternité	##/#### month/year		
REC-PCIME	##/#### month/year	Facility manager	
E-SantéCom	##/#### month/year		
NetSIGL 2.0	##/#### month/year		
FIS	##/#### month/year		
E-Gratuité	##/#### month/year		
Q24 : How many agents have been trained on MDE tools?			
**E-Qualité**			
State-registered nurses (IDE)	##		
Registered nurses (IB)	##		
Community health workers (AIS)	##		
Midwife/Maieutician (SFE/ME)	##		
Certified midwife (AB)	##		
Auxiliary midwife (AA)	##		
Manager of Essential Generic Medicines Depot (DMEG)	##		
Pediatric health attaché	##		
General physician	##		
CBHW	##		
Other (specify)	##		
**E-Flux financier**			
State-registered nurses (IDE)	##		
Registered nurses (IB)	##		
Community health workers (AIS)	##		
Midwife/Maieutician (SFE/ME)	##		
Certified midwife (AB)	##		
Auxiliary midwife (AA)	##		
Manager of Essential Generic Medicines Depot (DMEG)	##		
Pediatric health attaché	##		
General physician	##		
CBHW	##		
Other (specify)	##	Facility manager	
**REC- Maternité**			
State-registered nurses (IDE)	##		
Registered nurses (IB)	##		
Community health workers (AIS)	##		
Midwife/Maieutician (SFE/ME)	##		
Certified midwife (AB)	##		
Auxiliary midwife (AA)	##		
Manager of Essential Generic Medicines Depot (DMEG)	##		
Pediatric health attaché	##		
General physician	##		
CBHW	##		
Other (specify)	##		
**REC-PCIME**			
State-registered nurses (IDE)	##		
Registered nurses (IB)	##		
Community health workers (AIS)	##		
Midwife/Maieutician (SFE/ME)	##		
Certified midwife (AB)	##		
Auxiliary midwife (AA)	##		
Manager of Essential Generic Medicines Depot (DMEG)	##		
Pediatric health attaché	##		
General physician	##		
CBHW	##		

Other (specify)	*##*		
E-SantéCom			
State-registered nurses (IDE)	*##*		
Registered nurses (IB)	*##*		
Community health workers (AIS)	*##*		
Midwife/Maieutician (SFE/ME)	*##*		
Certified midwife (AB)	*##*		
Auxiliary midwife (AA)	*##*		
Manager of Essential Generic Medicines Depot (DMEG)	*##*		
Pediatric health attaché	*##*		
General physician	*##*		
CBHW	*##*		
Other (specify)	*##*		
NetSIGL 2.0	*##*		
State-registered nurses (IDE)	*##*		
Registered nurses (IB)	*##*		
Community health workers (AIS)	*##*		
Midwife/Maieutician (SFE/ME)	*##*		
Certified midwife (AB)	*##*		
Auxiliary midwife (AA)	*##*		
Manager of Essential Generic Medicines Depot (DMEG)	*##*		
Pediatric health attaché	*##*		
General physician	*##*		
CBHW	*##*		
Other (specify)	*##*		
FIS			
State-registered nurses (IDE)	*##*		
Registered nurses (IB)	*##*		
Community health workers (AIS)	*##*		
Midwife/Maieutician (SFE/ME)	*##*		
Certified midwife (AB)	*##*		
Auxiliary midwife (AA)	*##*		
Manager of Essential Generic Medicines Depot (DMEG)	*##*		
Pediatric health attaché	*##*		
General physician	*##*		
CBHW	*##*		
Other (specify)	*##*		
**E-Gratuité**			
State-registered nurses (IDE)	*##*		
Registered nurses (IB)	*##*		
Community health workers (AIS)	*##*		
Midwife/Maieutician (SFE/ME)	*##*		
Certified midwife (AB)	*##*		
Auxiliary midwife (AA)	*##*		
Manager of Essential Generic Medicines Depot (DMEG)	*##*		
Pediatric health attaché	*##*		
General physician	*##*		
CBHW	*##*		
Other (specify)	*##*		
Q25: How many financial, material, and medication audits has the DRSHP team carried out for ECDs over the last six months?	*##*	*RAF, Pharmacie district*	
Q26 : How many financial, material, and medication audits are carried out by the DRSHP team on behalf of the CMAs?	*##*	*Gestionnaire CMA*	
Q27 : Does your HF have resources or budget lines in their action plan to ensure the maintenance or replacement/renewal of equipment (tablets, accessories)?	*Yes/No*	*Facility manager*	
If not, why not? (Give 2 to 3 reasons)	*Text*		
Q28 : Does your FS have a power source?	*Yes/No*		
If not, why not? (Give 2 to 3 reasons)	*Text*		

If so, what is the source?	*Plaque solaire/ SONABEL/ Goupe electrogène/ Autre*		*(Multiple choice)*
Q29 : Does your FS have functional rolling logistics for the advanced strategy?	Yes/No		
If not, why not? (Give 2 to 3 reasons)	*Text*		
Q30 : Which MDE tool are you promoting?	*E-Qualité/E-gratuité/.....*	*ONG/MSHP*	Multiple tools can be selected. Set by tool
Q31 : How much does development cost?	### ### ###		
Q32 : How much does it cost to purchase the equipment?	### ### ###		
Q33 : How much does staff training cost?	### ### ###		
Q34 : How much does deployment and implementation cost?	### ### ###		
Q35 : How much does maintenance and technical support cost?	### ### ###		
Q36 : How much does supervision/coaching cost?	### ### ###		
**Identify the technological factors influencing the use of MDE tools (tool malfunctions, internet connection problems)**			
Q37 : Is your HF covered by the internet network?	Yes/No	*Facility manager*	
Q38 : Does your HF have the resources to purchase an internet subscription?	Yes/No		
**Identify the technological factors influencing the use of MDE tools (tool malfunctions, internet connection problems)**			
Q39 : For each of the MDE tools, how many days did you experience a malfunction during the last month? (If less than 24 hours, consider it one day.)		*Facility manager*	
E-Qualité	##		
E-Flux financier	##		
REC-Maternité	##		
REC-PCIME	##		
E-SantéCom	##		
NetSIGL 2.0	##		
FIS	##		
E-Gratuité	##		
Q40 : What is the nature of the malfunction observed?		*Facility manager*	
E-Qualité	Text		
E-Flux financier	Text		
REC-Maternité	Text		
REC-PCIME	Text		
E-SantéCom	Text		
NetSIGL 2.0	Text		
FIS	Text		
E-Gratuité	Text		
Q41 : Did repairing the malfunction require external intervention outside your HF?		*Facility manager*	
E-Qualité	Yes/No		
E-Flux financier	Yes/No		
REC-Maternité	Yes/No		
REC-PCIME	Yes/No		
E-SantéCom	Yes/No		
NetSIGL 2.0	Yes/No		
FIS	Yes/No		
E-Gratuité	Yes/No		
**Analyze the user experience with MDE, including tool acceptability and adoption, ease of use, stakeholder satisfaction, and determining factors (energy source, human resources)**			
Q42 : Which MDE tool do you use? (If you use multiple tools, ask each of the questions in sections 3.4 and 3.6 for each tool)	REC-Maternité ☐ / REC-PCIME ☐ / NetSIGL 2.0 ☐ / E-gratuité ☐ / E-Qualité ☐ /E-flux-Financier ☐ / FIS ☐ / E-SantéCom ☐ / None ☐	*All users*	
Q43: Do you think that the *[name of tool]* has made the work of healthcare staff easier?	*Yes/No/unknown*		
If not, give two or three reasons	*1………………………. 2……………………………… 3……………………………*		
Q44 : Do you find that the REC-Maternité tool facilitates consultations/post-abortion care/childbirth/CPN/prenatal and postnatal care?	*Yes/No*		Only REC-Maternity
If not, give two or three reasons.	*1………………………. 2……………………………… 3……………………………*		
Q45: Do you find that the REC-PCIME tool facilitates curative consultations for children?	*Yes/No*		Only REC-PCIME
If not, give two or three reasons	*1………………………. 2……………………………… 3……………………………*		
Q46 : Do you find that the E-gratuité tool facilitates the transmission of reports?	*Yes/No*		Only E-gratuité
If not, give two or three reasons.	*1………………………. 2……………………………… 3……………………………*		
Q47: Do you find that the E-Quality tool facilitates healthcare quality management?	*Yes/No*		OnlyE-

			Qualité
If not, give two or three reasons.	*1………………………. 2……………………………. 3……………………………*		
Q48: Do you find that the E-flux-Financier tool optimizes financial management?	*Yes/No*		Only E-flux-Financier
If not, give two or three reasons	*1………………………. 2……………………………. 3……………………………*		
Q49: Do you find that the FIS tool optimizes the management of free invoices?	*Yes/No*		Only FIS
If not, give two or three reasons.	*1………………………. 2……………………………. 3……………………………*		
Q50: Do you think that the E-SantéCom tool improves CBHW services?	*Yes/No*		Only E-SantéCom
If not, give two or three reasons.	*1………………………. 2……………………………. 3……………………………*		
Q51: Do you find that the tool [*name of tool*] requires extra effort on your part?	*Yes/No/Unknown*		
If so, give 2 or 3 examples	*1………………………. 2……………………………. 3……………………………*		
Q52: Do you think that most of your colleagues use at least one MDE tool?	*Yes/No/Unknown*		
If not, give two or three reasons.	1*1………………………. 2……………………………. 3……………………………*		
Q53: Do you think that rolling out the tool nationwide will have a positive impact on Burkina Faso's healthcare system?	*Yes/No/Unknown*		
If so, give two or three examples of positive impacts	*1………………………. 2……………………………. 3……………………………*		
If not, give two or three reasons.	*1………………………. 2……………………………. 3……………………………*		
When using REC-Maternité and E-flux-Financier (When using multiple tools, ask each of the questions in sections 3.4 and 3.6 for each tool)			
Q54: How do you rate the use of the [tool] in the context of MDE?	*Very difficult ☐/Difficult ☐/ Easy ☐/Very Easy ☐*		
If very difficult or difficult, list two or three reasons	*1………………………. 2……………………………. 3……………………………*		
Q55: Have you received formal training in the use of the [tool] in the context of MDE?	*Yes ☐/No ☐*		
Q56: When did this formal training take place?	*Months and years*		
Q57: How long did this training last?	|__|__| *Days*		
Q58: What do you think about the length of formal training?	*Too long ☐/ Correct☐/ Too short ☐*		
Q59: At the end of this formal training, I find that...			
- The materials used for the training were:	*0- Not at all Satisfactory ☐/1- Not Satisfactory ☐/2- Satisfactory ☐/3- Very Satisfactory ☐*		
List two or three points of dissatisfaction.	*1………………………. 2……………………………. 3……………………………*		
- The training was facilitated by:	*0- Not at all Satisfactory ☐/1- Not Satisfactory ☐/2- Satisfactory ☐/3- Very Satisfactory ☐*	*All users*	
List two or three points of dissatisfaction	*1………………………. 2……………………………. 3……………………………*		
- The level of the trainers was:	*0- Not at all Satisfactory ☐/1- Not Satisfactory ☐/2- Satisfactory ☐/3- Very Satisfactory ☐*		
List two or three points of dissatisfaction	*1………………………. 2……………………………. 3……………………………*		
Q60: Did the formal training meet your expectations?	*Yes ☐/No ☐*		
If not, list two or three reasons	*1………………………. 2……………………………. 3……………………………*		
Q61: After the formal training, did I acquire the knowledge necessary to use the tool?	*0- Completely disagree. ☐/1- Disagree ☐/2- Agree ☐/3- Completely agree ☐*		
If you disagree or strongly disagree, what limitations remain?	*1………………………. 2……………………………. 3……………………………*		
Q62: Are you comfortable using the tool after formal training?	*Yes ☐/No ☐*		
If not, list two or three reasons	*1………………………. 2……………………………. 3……………………………*		
List two or three areas for improvement in this formal training	*1………………………. 2……………………………. 3……………………………*		
Q63: Have you received peer training (informal) on how to use the tool in the context of MDE?	*Yes ☐/No ☐*		
Q64: How long did this training last?	|__|__| *Jours*		
Q65: What do you think about the length of the peer training?	*Too long ☐/ Correct☐/ Too short ☐*		
Q66: Compared to peer training, I am:	*0- Not at all satisfied ☐/1- Not satisfied ☐/2- Satisfied ☐/3-Very Satisfied ☐*		
List two or three points of dissatisfaction	*1………………………. 2……………………………. 3……………………………*		
Q67: Did peer training meet your expectations?	*Yes☐/No ☐*		
If not, please explain why.	*…………………………………………………………………………………………*		
Q68: After peer training, did I acquire the knowledge necessary to use the tool?	*0- Completely disagree. ☐/1- Disagree ☐/2- Agree ☐/3- Completely agree ☐*		
If you disagree or strongly disagree, what limitations remain?	*1………………………. 2……………………………. 3……………………………*	*All users*	
Q69: Are you comfortable using the tool after peer training?	*Yes ☐/No ☐*		
If not, why not?	*…………………………………………………………………………………………*		
Q70: "I am proficient or comfortable using the tool." Do you agree with this statement?	*0- Completely disagree. ☐/1- Disagree ☐/2- Agree ☐/3- Completely agree ☐*		
List two or three areas for improvement in the tool	*1………………………. 2……………………………. 3……………………………*		
Q71: How many times have you used the MDE tool?	*1-2 times ☐/3-4 times ☐/ 5-6 times ☐/ More than 6 times ☐*		
Q72: What is your attitude towards the MDE tool?	*Positive (Favorable) ☐/ Negative (Unfavorable) ☐*		
Q73: How satisfied are you with the tool?	*0- Not at all satisfied ☐/1- Not satisfied ☐/2- Satisfied ☐/3-Very Satisfied ☐*		
Q74: Does your HF organize care for the indigent, social cases, and patients without companions?	Yes/No	*Facility manager*	
If not, why not? (Give 2 to 3 reasons)	Text		
Q75: Does your HF organize continuing education refresher courses?	Yes/No	*Facility manager*	

If not, why not? (Give 2 to 3 reasons)	Text		
Q76: Does your HF apply financial management procedures (availability of financial management texts and procedures)?	Yes/No	*Facility manager*	
If not, why not? (Give 2 to 3 reasons)	Text		
Q77: Does your HF pay its suppliers for essential generic drugs on time?	Yes/No		
Q78: Does your HF pay its cleaning service providers on time?	Yes/No		
Q79: Does your HF pay its security service providers on time?	Yes/No		
Q80: Does your HF have a manual for managing stocks of medicines and consumables?	Yes/No	*Facility manager*	
If yes, management manual seen	Yes/No		
If not, why not? (Give 2 to 3 reasons)	Text		
Q81: Does your HF have tools for managing stocks of medicines and consumables?	Yes/No		
Q82: Does your HF have a functional power backup source?	Yes/No	*Facility manager*	
If not, why not? (Give 2 to 3 reasons)	Text		
Q83: Does your HF have a maintenance system for energy source installations?	Yes/No		
Q84: Did your HF experience an energy outage last month?	Yes/No		
Q85: Does your HF provide maintenance for hardware and equipment?	Yes/No	*Facility manager*	
Q86: Does your HF have an up-to-date dashboard of recommendations and their implementation plan?	Yes/No	*Facility manager*	
Q87: Does your HF have a plan for monitoring the implementation of the recommendations?	Yes/No		
Q88: Does your HF document the actions taken in response to the recommendations?	Yes/No		
Q89: Does your HF have reference and cross-reference sheets?	Yes/No	*Facility manager*	
Q90: Does your HF have a procedure and tools for managing user rejection of references?	Yes/No		
Q91: Does your HF have a room for the EPI and a cold chain?	Yes/No	*Facility manager*	
Q92: Does your HF have a vaccination schedule?	Yes/No		
Q93: Does your HF have a system for locating people who have lost touch?	Yes/No		
If so, give an example	Text		
Q94: Does your HF have a system for collecting complaints and suggestions from users?	Yes/No	*Facility manager*	
Q95: Does your HF have a system for managing user complaints and suggestions?	Yes/No		

Supplementary Table 1: Multicollinearity test (VIF) for the six multivariate models

Independent variables (Predictors)	Service Delivery		Community health management		Pharmaceutical supply management		Quality of care management		Financial flow management		Free healthcare management	
	VIF	1/VIF	VIF	1/VIF	VIF	1/VIF	VIF	1/VIF	VIF	1/VIF	VIF	1/VIF
**Sociodemographic**												
Sex	0.56	1.785	1.16	0.864	1.35	0.742	0.72	1.394	N/A	N/A	2.89	0.346
Age group	0.58	1.714	1.26	0.792	N/A	N/A	0.79	1.273	0.37	2.725	N/A	N/A
Education level	0.17	5.780	N/A	N/A	2.14	0.466	N/A	N/A	0.99	1.013	N/A	N/A
Professional experience	1.18	0.849	N/A	N/A	2.82	0.355	N/A	N/A	0.33	2.990	0.40	2.485
Literacy	N/A	N/A	N/A	N/A	N/A	N/A	N/A	N/A	N/A	N/A	N/A	N/A
**Organizational/Contextual**												
Distance to health facility	N/A	N/A	N/A	N/A	N/A	N/A	N/A	N/A	N/A	N/A	N/A	N/A
Level of care	N/A	N/A	N/A	N/A	N/A	N/A	N/A	N/A	N/A	N/A	N/A	N/A
District	N/A	N/A	1.98	0.505	N/A	N/A	0.93	1.073	N/A	N/A	N/A	N/A
Energy availability	N/A	N/A	N/A	N/A	N/A	N/A	N/A	N/A	0.20	4.898	0.36	2.771
Internet availability	N/A	N/A	N/A	N/A	N/A	N/A	N/A	N/A	0.26	3.852	N/A	N/A
Tablets availability	0.38	2.653	1.30	0.772	1.75	0.572	N/A	N/A	0.43	2.341	N/A	N/A
Formal training	0.14	7.238	N/A	N/A	N/A	N/A	N/A	N/A	N/A	N/A	5.92	0.169
Peer training	N/A	N/A	N/A	N/A	N/A	N/A	N/A	N/A	N/A	N/A	0.56	1.780
Ease of use	0.34	2.985	0.42	2.361	N/A	N/A	N/A	N/A	0.27	3.771	0.41	2.418
Mastery of the tool	0.14	7.279	N/A	N/A	N/A	N/A	N/A	N/A	0.62	1.605	1.76	0.567
Attitude towards the tool	N/A	N/A	N/A	N/A	N/A	N/A	N/A	N/A	N/A	N/A	N/A	N/A
**Mean VIF**	**0.44**		**1.22**		**2.01**		**0.81**		**0.43**		**1.76**	

Note : *A VIF (Variance Inflation Factor) value < 5 indicates the absence of problematic multicollinearity.* ***N/A*** *(Not Applicable) indicates variables that were not included in the specific multivariate model based on univariate pre-selection (p < 0.20)*
